# Age and axial length on peripapillary retinal nerve fiber layer thickness measured by optical coherence tomography in nonglaucomatous Taiwanese participants

**DOI:** 10.1371/journal.pone.0179320

**Published:** 2017-06-08

**Authors:** Pai Huei Peng, Sheng Yao Hsu, Wei Shin Wang, Mei Lan Ko

**Affiliations:** 1 Department of Ophthalmology, Shin-Kong Wu Ho-Su Memorial Hospital, Taipei, Taiwan; 2 Department of Ophthalmology, China Medical University Hospital- An Nan Branch, Tainan, Taiwan; 3 Antibody Engineering Technology Department, Biomedical Technology and Device Research Laboratories, Industrial Technology Research Institute, Chutung, Hsinchu, Taiwan; 4 Department of Ophthalmology, National Taiwan University Hospital, Hsin Chu Branch, Hsinchu City, Taiwan; 5 Department of Biomedical Engineering and Environmental Science, National Tsing Hua University, Hsinchu City, Taiwan; Bascom Palmer Eye Institute, UNITED STATES

## Abstract

**Purpose:**

This study investigates the influence of age and axial length (AL) on the peripapillary retinal nerve fiber layer (RNFL) thickness, as measured by optical coherence tomography (OCT).

**Methods:**

Healthy patients visiting an eye clinic at a county hospital were recruited. All participants underwent comprehensive ophthalmologic examinations, and their retinas were scanned using 3D OCT-1000. In total, 223 patients with 446 eyes were included. The mean age and AL were 42.07 ± 13.16 (21–76) years and 25.38 ± 1.73 (21.19–30.37) mm, respectively.

**Results:**

The average RNFL thickness decreased by 2.71 μm for every 10-year increase in age (*P* < 0.001). Age-related RNFL thinning was more significant in participants older than 41 years (-0.24μm/year; *P* = 0.015). The earliest sector showing a significant decline in RNFL thickness was after 35 years of age (-0.70μm/year; *P* = 0.011) at the superior quadrant and at the 1–2 o’clock hour (-1.42μm/year; *P* = 0.009). Meanwhile, the maximal rate of age-associated RNFL decay was observed in these two regions as well. The reduction of RNFL with age progression did not differ in eyes with long AL (> 27 mm; -0.16μm/year) or those with short AL (< 25 mm; -0.22μm/year). For every 1-mm-greater AL, RNFL was thinner by 1.78 μm (*P* < 0.001). The inferior quadrant showed the greatest tendency of RNFL decline with longer AL (4.46 μm/mm; *P* < 0.001).

**Conclusions:**

The factors of age and AL should be considered when interpreting the results. Significantly age-associated RNFL thinning was found in participants older than 41 years. Reduction of RNFL thickness with increasing age was not affected by AL. Topographic variations in RNFL thinning were observed in that the maximal decline of RNFL thickness with advancing age at the superior quadrant whereas with elongation of AL at the inferior quadrant.

## Introduction

Glaucoma, a specific form of optic neuropathy with irreversible visual loss, affects millions of people worldwide. The progressive loss of the peripapillary retinal nerve fiber layer (RNFL) and the deeper and larger optic disk cup are recognized as pathological alterations of glaucoma. Therefore, the assessment of RNFL plays a major role in the diagnosis and follow-up of glaucoma[1, 2]. Compared with conventional red-free photography, optical coherence tomography (OCT), which measures RNFL thickness based on reflectivity changes between adjacent tissues, provides instant and quantitative information on RNFL thickness. However, the performance of OCT could be compromised by several factors such as image quality[3], accuracy of the centering of the scan circle [4], and the influence of myopia[5, 6].

The incidence of myopia in Asian countries is extremely high [7], which includes Taiwan[8]. The elongation of axial length (AL) occurs because increased negative refractive power and certain optic disk features of myopic eyes affect the performance accuracy of OCT[9–11]. The reported associations between AL and RNFL thickness vary with different study populations, OCT instruments, and whether ocular magnification is adjusted. Certain studies have shown that the average RNFL thickness inversely correlated with AL or negative refractive power[2, 12, 13]. By contrast, certain studies have reported a positive relationship between AL and RNFL thickness [14, 15].

Although most studies have been in agreement that RNFL thickness decreased with age [2, 13, 16, 17], distinguishing between age- or glaucoma-induced RNFL thinning is still a challenge at this point. A longitudinal study spanning for 4 years on 192 eyes found approximately 0.2% per year of age-related thinning in circumpapillary and macular RNFL [18]. The 3D OCT-1000 is a spectrum-domain OCT that uses high-resolution raster scan patterns, and is designed without correction for age and ocular magnification. Information regarding demographic and ocular parameters that affect RNFL measurement by 3D OCT-1000 are limited. Further knowledge regarding the factors contributing to misleading OCT findings is critical, because the determination of glaucomatous damage by OCT is to compare our findings with the normative database developed by manufacturers. The effects of age and refraction on the measurement of peripapillary RNFL thickness by 3D OCT-1000 were studied. In a similar manner, the topographic profile of RNFL thickness in myopic eyes was assessed.

## Materials and methods

Healthy patients were invited to participate in the study when they visited an eye clinic at a county hospital from April to December of 2011. The IRB Number (HCGH99IRB-8) was specifically approved by the institution Review Board of Hsin-Chu General Hospital. Informed consent was obtained from each participant, and the tenets of the Declaration of Helsinki were followed. Detailed ophthalmic examinations, including refractive error measurement conducted using the Autoref keratometer RC-5000 (Tomey Co. Nagoya, Japan), slit lamp examination, intraocular pressure (IOP) measurement using the Non-Contact Tonometer NT-530 (Nidex Co., Gamagori, Japan), fundoscopy, and a visual field (VF) test by using an Octopus Visual Field Analyzer (Interzeag AG, Berne, Switzerland), were performed. AL measurements were obtained using IOL Master (Carl Zeiss MEDITEC, Dublin, USA). Participants who were contraindicated for pupillary dilation or intolerable to topical anesthetics or mydriatics were excluded. Only paired eyes of participants were considered for analyses, unless any of the eyes exhibited any corneal, retinal, or optic nerve diseases, a best corrected visual acuity of less than 0.6, IOP ≥ 22 mmHg, or unreliable VF results (false-positive and false-negative rate > 15%, fixation losses > 20%). Retina scanning by 3D OCT-1000 (Topcon, Japan) with a 512 × 128-fast scan protocol was performed, which used a 3.4-mm diameter circle around the optic nerve head and covered a 6 × 6 mm^2^ area. The image volume consists of 128 frames each with 512 A-lines. For data processing and analysis, the optic disc scans were segmented using the Topcon Advanced Boundary Segmentation (TABS) algorithm in FastMapTM 8.11. In brief, a circumpapillary annulus of 1.7 mm radius was extracted from the 3D volume data sets to generate retinal NFL thickness measurements corresponding to the overall circle as well as to the individual temporal, superior, nasal, and inferior quadrants. All optic disc scans were automatically centered for the circumpapillary analyses. To avoid centering errors, two experienced technicians checked and adjusted positions of the scan by placing a transparent plastic plate with multiple concentric rings on red free fundus images. Eyes were also excluded if image quality of OCT less than 45 or any algorithm line failures. The three-dimensional data setting to generate RNFLT measurements corresponding to the overall circle, the individual quadrants (temporal, superior, nasal, inferior) and 12 clock hours were presented.

To determine the break-point that the RNFL showed a significant decline with age, the rates of thinning before and after that age were compared (started from age 21 and up) until the significant difference was disclosed.

Statistical analyses were performed with R program software. The generalized estimating equation (GEE) linear model was used to examine the association between RNFL thickness and age, AL, and spherical equivalent (SE), and the inter-eye correlation was taken into account. A *P* value < 0.05 was considered statistically significant.

## Results

The study population consisted of 223 healthy participants (446 eyes) aged 21–79 years. Of these participants, 104 (42.1%) were men. Table 1 shows the clinical characteristics of the study population. The right eyes are more myopic than the left eyes (p = 0.01).

**Table 1:** Age and biometric features of the study participants.

	Parameter	Age (years)	SE^b^ (Diopter)	AL^c^ (mm)
OU	Mean ± SD^a^	42.07 ± 13.16	-4.64 ± 4.11	25.38 ± 1.73
	Range	21 ~ 76	-17.50 ~+4.25	21.19 ~ 30.37
OD	Mean ± SD^a^		-4.75±4.16	25.37±1.75
	Range	same	-17.25~+4.25	21.19 ~ 29.91
OS	Mean ± SD^a^		-4.53±4.06	25.38±1.72
	Range	same	-17.25~+4.25	21.44 ~ 30.37

^a^SD, standard deviation.

^b^SE, spherical equivalent.

^c^AL, axial length.

In agreement with most previous studies, our results revealed that the average RNFL thickness decreased with age. Overall, for every 10-year increase in age, the average RNFL thickness decreased in the magnitude of 2.71 μm (*P* < 0.001). Age-related global RNFL thinning was more pronounced after 41 years of age (rate of thinning: 0.24 μm/year; 97.5% CI, 0.05 to 0.44; *P* = 0.015).The right eye showed earlier thinning than the left eye, however there is no significant difference of the rate of RNFL thickness in paired eyes under Dummy variable analysis (p = 0.121). Sectoral variations were noticed in age-related RNFL thinning (Table 2), with the superior quadrant displaying the earliest as well as the steepest decline in participants after 35 years of age (rate of thinning: 0.70 μm/year; 97.5% CI, 0.16 to 1.25; *P* = 0.011), followed by the temporal quadrant (age of 41 years; rate of thinning: 0.36 μm/year; 97.5% CI, 0.06 to 0.67; *P* = 0.020). RNFL thickness at the inferior and nasal quadrants was unaffected by age.

**Table 2:** Age when thinning of mean and quadrant RNFL thickness was significantly different.

	OU^a^				OD^b^				OS^c^			
	Age	Rate of thinning (μm/year)	97.5% CI^d^	P	Age	Rate of thinning (μm/year)	97.5% CI	P	Age	Rate of thinning (μm/year)	97.5% CI	P
Overall	41	0.24	0.05–0.44	0.015	41	0.31	0.04–0.58	0.024	50	0.18	0.01–0.34	0.036
Superior	35	0.70	0.16–1.25	0.011	36	0.81	0.2–1.42	0.010	38	0.64	0.06–1.21	0.031
Nasal		0.07	-0.15–0.02	NS^e^		0.07	-0.21–0.05	NS		0.05	-0.18–0.07	NS
Inferior		0.23	0.12–0.34	NS		0.22	0.05–0.40	NS		0.22	0.07–0.38	NS
Temporal	41	0.36	0.06–0.67	0.020	41	0.49	0.02–0.95	0.040	54	0.25	0.03–0.47	0.027

^a^OU, bilateral eyes

^b^OD, right eye

^c^OS, left eye

^d^CI, confidence interval

^e^NS, no significance

**Fig 1:**
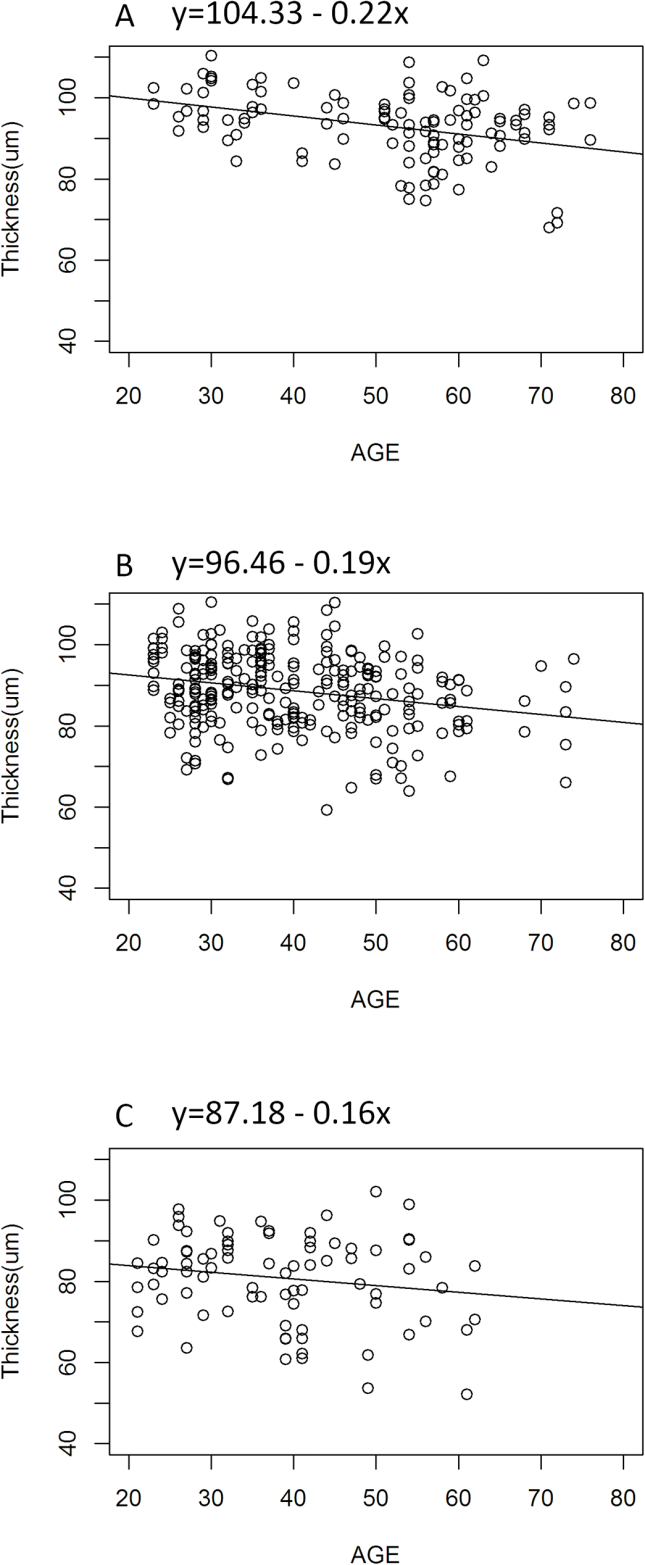
The rates of age-related RNFL thinning in different axial length groups. Liner model was applied to explore the relationship between AL and the rates of age-related RNFL thinning. (A) Short AL group with AL < 25 mm. (B) Medium AL group with AL 25 to 27 mm. (C) Long AL group with AL > 27 mm. No significant difference was shown in any of these groups. AL, axial length; T, retinal nerve fiber layer thickness.

The study eyes were further divided into three groups according to the AL: short (< 25mm), medium (25 to 27 mm), and long (> 27 mm) AL group. The rates of age-related reduction of RNFL thickness in these three groups were -0.22 (short AL group), -0.19 (medium AL group), and -0.16 (long AL group) μm/year, respectively (Fig 1). The geepack are used for GEE models in R. There exists no significant differences among the following each two groups: long and short AL groups (P = 0.86), long and medium AL groups (P = 0.84) and short and medium AL groups (P = 0.53).

Table 3 shows the results regarding age when a significant correlation with clock hour RNFL thickness was noticed. For the right eye, the earliest age-related RNFL thinning was found after 35 years at 1–2 o’clock hour (1.89 μm/year; 97.5% CI, 0.67 to 3.11; *P* = 0.003). In addition, the maximal rate of age-associated RNFL thinning was located at this clock-hour. The other regions showing a significant age-related RNFL thinning were at 12–1 (after age 44 with a rate of thinning 0.49μm/year; 97.5% CI, 0.01 to 0.96; *P* = 0.044), 8–9 (after age 40 with a rate of thinning 0.77μm/year;; 97.5% CI, 0.05 to 1.49; *P* = 0.037), and 11–12 o’clock (after age 39 with a rate of thinning 0.74μm/year; 97.5% CI, 0.11 to 1.37, *P* = 0.021), respectively. For the left eye, the earlier age-related RNFL decline was found after 38 years at 12–1 o’clock (0.83 μm/year; 97.5% CI, 0.01 to 1.65; *P* = 0.048). The other regions showing a significant RNFL thinning as progression of age were at 1–2 (after age 41 with a rate of thinning 0.69μm/year; 97.5% CI, 0.02 to 1.36; *P* = 0.044), 2–3 (after age 55 with a rate of thinning 0.29μm/year; 97.5% CI, 0.02 to 0.57; *P* = 0.038), 4–5 (after age 48 with a rate of thinning 0.45μm/year; 97.5% CI, 0.04 to 0.87; *P* = 0.034), and 11–12 o’clock (after age 49 with a rate of thinning 0.45μm/year; 97.5% CI, 0.02 to 0.88; *P* = 0.041), respectively. The locations of RNFL thinning in clock hour were in agreement with the results of quadrant analysis.

**Table 3:** Age when thinning of clock hour RNFL thickness was apparent.

Clock hour	OD				OS			
	Age	Rate of thinning (μm/year)	97.5% CI	P	Age	Rate of thinning (μm/year)	97.5% CI	P
12–1	44	0.49	0.01–0.96	0.044	38	0.83	0.01–1.65	0.048
1–2	35	1.89	0.67–3.11	0.003	41	0.69	0.02–1.36	0.044
2–3		-0.05	-0.26–0.14	NS	55	0.29	0.02–0.57	0.038
3–4		-0.08	-0.20–0.04	NS		0.23	0.10–0.37	NS
4–5		0.08	-0.23–0.06	NS	48	0.45	0.04–0.87	0.034
5–6	37	0.85	0.04–1.66	0.039	54	0.42	0.09–0.75	0.014
6–7	36	1.46	0.32–2.60	0.013	37	0.98	0.01–1.95	0.047
7–8		0.47	0.23–0.72	NS		0.05	-0.15–0.25	NS
8–9	40	0.77	0.05–1.49	0.037		0.08	-0.24–0.08	NS
9–10		0.25	0.09–0.40	NS		0.05	-0.17–0.07	NS
10–11		0.31	0.13–0.49	NS		-0.03	-0.20–0.13	NS
11–12	39	0.74	0.11–1.37	0.021	49	0.45	0.02–0.88	0.041

The average RNFL thickness decreased with eyeball elongation in the magnitude of 1.78 μm per 1-mm increase in AL (*P* < 0.001). In addition, RNFL thickness revealed regional differences associated with AL. The most significant thinning of RNFL associated with a longer AL was found at the inferior quadrant, at a rate of 4.46 μm/mm (*P* < 0.001). RNFL thickness decreased by 1.89 (*P* = .031) and 1.70 μm (*P* = 0.010) at the superior and nasal quadrants with a 1-mm increase in AL. No significant correlation was observed between the temporal quadrant RNFLs and AL.

The spherical equivalent was highly correlated with AL (R^2^ = 0.863 in this study); therefore, we only considered the effects of AL on RNFL thickness in this study.

## Discussion

In the current study used 3D OCT-1000 to measure RNFL thickness, we found that the average RNFL thickness decreased with longer AL as well as with advancing age, particularly after 46 years of age. The magnitude of reduction in RNFL thickness with increasing age was similar in eyes with longer or shorter AL. Meanwhile, RNFL thinning with AL and age did not occur in a uniform manner around the optic disc.

Thinner RNFL measurements by OCT associated with advancing age have been studied extensively. For every 10-year increase in age, decreases in the average RNFL thickness ranged from 1.5 to 2.5 μm[2, 13, 16, 17]. Histological data have also revealed the loss of optic nerve fibers with age [19, 20]. Approximately 4000–5000 optic nerve fibers are lost per year[20]. The reduction in RNFL thickness with increasing age resulted mainly from the decrease of neuronal and glial elements, rather than from vascular components, as suggested by a recent morphological study[21]. Our results also revealed that the age-related RNFL thinning rate was statistically significant when patients reached a certain age. This finding was in agreement with that reported by Parikh et al. [16]. In their study, 187 participants (one eye of each participant was selected for analysis) were divided by age into 4 groups: 5–20, 20–35, 35–50, and >50 years, and the authors reported that age-associated RNFL thinning was most prominent after the age of 50 years. Quigley et al. provided histological data that the RNFL thinning rate was faster after 50 years of age (approximately 2500 optic nerve fibers lost per year before the age of 50 years vs 7500 lost per year after the age of 50 years), although the authors examined only 5 human eyes[22]. The possible reasons why our results of significant age-related RNFL thinning is younger (41 years old) than the prior two studies might due to further detailed statistical analysis by each year and criteria for selecting of studied population (refractive error within ± 5 diopter of sphere and 3 Diopter cylinder by Parikh et al[16]. In clinical practice, Feuer et al. suggested that to confirm the loss of RNFL caused by glaucoma, the rate of change must be faster than the expected rate from the 95% confidence interval at a particular location [17].

In agreement with other studies, our results also showed a topographic variation in age-related RNFL thinning[13, 16, 17]. Studies have reported that the maximal rate of decay in RNFL was at the superior quadrant, whereas the inferior region appeared to be the most resistant sector to RNFL loss[16, 17]. The present study was in agreement in terms of the superior sector having had the maximal reduction of RNFL thickness, and that it was the earliest region exhibiting RNFL thinning (by the age of 35 years). The reason for a topographic difference in age-related RNFL thinning remains unknown. Jonas et al. found that age-associated axonal loss particularly affected small nerve fiber axons [20]. FitzGibbon and Taylor recently mentioned that retinal ganglion cell axons in the inferior and nasal quadrants were on average larger than those in the superior and/or temporal quadrants[23]. Additional evidence is required to confirm whether small nerve axons in the superior quadrant are more vulnerable to age-related thinning. The present study also showed aging difference on the average RNFL between both eyes. The differences between both eyes of the same individual are commonly noted in clinic, for instance, refractive error, cataract stage, intraocular pressure and blood flow. The dominant eye may consume more oxygen than another eye due to working harder and sending more visual messages to the brain. These various factors may influence the aging difference between both eyes. Further studies are needed to reconfirm and clarify the patho-mechanisms.

A positive or negative correlation between the average RNFL thickness and AL measured by OCT was reported. The default AL in TOPCON 3D-OCT is 24.39 mm, and the average refractive error of Asian subjects for the normative database is -0.66 ± 1.70 D (range: -5.75 to 2.88 D). In this present study, the mean AL and refractive error is 25.36 mm and -4.68 ± 4.16 D (range: -17.5 to +4.25 D), respectively. Due to a considerable proportion of myopic eyes in our study population, the mean RNFL thickness (88.17 ± 10.61 μm) is lower than other studies on healthy Chinese eyes (107.02 μm in Hsu study and 96.04 μm in Qu study[15, 24]. As a result, to avoid over-diagnosis of glaucoma, knowledge of the effects of AL on RNFL is warranted, especially for high myopic eyes. Increased AL leading to a larger retinal surface, while with unchanged numbers of retinal ganglion cells axons could cause thinner RNFL measured by OCT related to increased AL. To date, no anatomical evidence has been presented showing that retinal ganglion cells/axons degeneration is due to the elongation of AL.

Although both age and AL factors affect the measurement of RNFL by OCT, eyes with longer AL did not show a faster rate of age-associated RNFL thinning than those with shorter AL. Another point is that the RNFL is rather thin in aged high myopic people; however, their VF results remain within normal range. Further studies of any impacts of the reduced RNFL thickness on visual functions are needed to determine.

In addition to the reduction in the average RNFL thickness in myopic eyes, a variation in the RNFL profile with increasing AL has been reported[5, 14, 15, 25]. A study included 115 eyes of 115 healthy participants (75 eyes with high myopia and 40 with low-to-moderate myopia), and reported that the most frequent abnormal sector of RNFL thickness was at the 2 o’clock position ^5^, which differed from our study, and thus, showed that the effect of AL on RNFL thickness was more pronounced in the inferior quadrant. In the Kim et al's study, of the 48 myopic patients, they reported thinner RNFL's in the higher myopia group than those in both lower and moderate myopia groups in the non-temporal quadrants; however, while in the temporal quadrant, thicker RNFL's are associated with the higher myopia group [25]. In the present study, although we did not find a significant correlation between AL and the RNFL at the temporal quadrant, the RNFL decreased with increasing AL at non-temporal quadrants. Variations in sample composition might be responsible for these discrepancies.

This study has a number of limitations, and thus, additional comprehensive research is warranted. First, the participants were recruited from a clinic; therefore, selection bias cannot be denied. Second, within the constraints of a cross-sectional design, our findings might be inaccurate in estimating the longitudinal changes of RNFL.

In conclusion, RNFL thickness measured by 3D OCT-1000 is reduced by 2.71 μm per decade with progression of age, and by 1.78 μm per 1-mm increase in AL. Decline of RNFL thickness is significantly beyond the age of 41 years. The superior quadrant shows the earliest as well as the maximal slope of age-related RNFL thinning. On the other hand, the most severely RNFL thinning with increasing AL is seen at the inferior quadrant. The rates of age-associated RNFL thinning are not different in eyes with long or short AL. Further studies are warranted to explore the reasons accounting for the regional variations of age- and AL- related decline in RNFL thickness.
